# Incidental discovery of unilateral hydronephrosis unveiling psoas major desmoid-type fibromatosis in a 24-year-old male: A case report with a 5-year follow-up

**DOI:** 10.1097/MD.0000000000039042

**Published:** 2024-07-26

**Authors:** Abdulrahman Almjersah, Habib Olaisheh, Rabab Salloum, Zuheir Alshehabi, Emad Almjersah

**Affiliations:** a Faculty of Medicine, Cancer Research Center, Tishreen University, Latakia, Syria; b Syrian Medical Research Group, Damascus, Syria; c Department of Pathology, Cancer Research Center, Tishreen University, Latakia, Syria; d Youssef Ali Surgical Hospital, Latakia, Syria.

**Keywords:** case report, desmoid-type fibromatosis, hydronephrosis, psoas muscle, retroperitoneum

## Abstract

**Rationale:**

Desmoid-type fibromatosis (DTF), also known as aggressive fibromatosis, is a rare neoplasm originating from the fascial or musculoaponeurotic tissues. While benign and characterized by slow growth, it exhibits local aggressiveness and lacks specific clinical characteristics. However, in a considerable percentage of patients, it could be asymptomatic and discovered by accident during routine clinical examinations. Only a few cases of DTF arising from the psoas major muscle have been reported in the medical literature.

**Patient concerns:**

A 24-year-old male, asymptomatic and without significant personal or family medical history, was diagnosed with grade 2 hydronephrosis by abdominal ultrasonography during a routine physical examination. This diagnosis was made 15 days after undergoing uncomplicated open-heart surgery to repair an atrial septal defect.

**Diagnosis:**

Intravenous pyelogram revealed hydronephrosis with dilation of the pelvicalyceal system. Ureteroscopy ruled out any intrinsic lesions of the ureter. Contrast-enhanced computed tomography identified a 3.5 × 2 × 5.2 cm mass in the retroperitoneum, closely associated with the psoas muscle and enveloping the ureter adjacent to the iliac artery. Postoperative pathological analysis confirmed a definitive diagnosis of sporadic DTF.

**Interventions:**

The patient underwent exploratory abdominal surgery, during which the tumor was resected without any intraoperative complications.

**Results:**

After close monitoring over a 5-year follow-up period, which included periodic physical examinations, magnetic resonance imaging, and ultrasonography, no local recurrence was detected.

**Lessons:**

Achieving an accurate preoperative diagnosis presents a challenge in cases involving retroperitoneal tumors originating from the psoas major muscle and encasing the ureter. However, the insertion of a double J stent is deemed a crucial step in the surgical process, facilitating the dissection and isolation of the ureter from the tumor while preserving kidney function.

## 1. Introduction

Desmoid-type fibromatosis (DTF) is a rare soft tissue tumor, comprising about 0.03% of all neoplasms,^[1]^ with an annual incidence ranging from 2.4 to 4.3 cases per million individuals.^[2]^ Although benign and nonmetastatic, it is locally aggressive and has notable potential for recurrence.^[3–5]^ While the exact etiology of DTF remains elusive, significant risk factors include a history of familial adenomatous polyposis (FAP) or Gardner syndrome, along with a history of surgical trauma. DTF predominantly affects young females, typically between the ages of 25 and 35 years.^[2,5,6]^

Patients with DTF typically lack a specific clinical presentation and may remain asymptomatic, with the tumor being incidentally discovered.^[3,4,7–9]^

While a watchful-wait strategy is now considered the go-to approach for managing patients with DTF, surgical intervention remains crucial, particularly in cases with complications.^[4,9–11]^

Herein, we present a case of an asymptomatic 24-year-old male patient with a desmoid tumor originating from the fascia of the psoas major muscle. The patient was found to have unilateral hydroureteronephrosis incidentally detected by ultrasonography (US) 15 days after uncomplicated open-heart surgery for atrial septal defect (ASD).

## 2. Case report

A 24-year-old male was referred to the urology clinic for further evaluation of grade 2 left-sided hydronephrosis detected by US. The patient was asymptomatic, and this finding was discovered incidentally during routine clinical examinations conducted 15 days after he had undergone uncomplicated open-heart surgery for an ASD via a midline incision.

The patient’s medical history was unremarkable, and he denied any history of trauma, abdominal surgery, or steroid hormonal treatment. Additionally, the patient’s family history was insignificant, and there was no evidence of familial diseases suggestive of Gardner syndrome, FAP, or any other genetic disorders.

**Figure 1.**
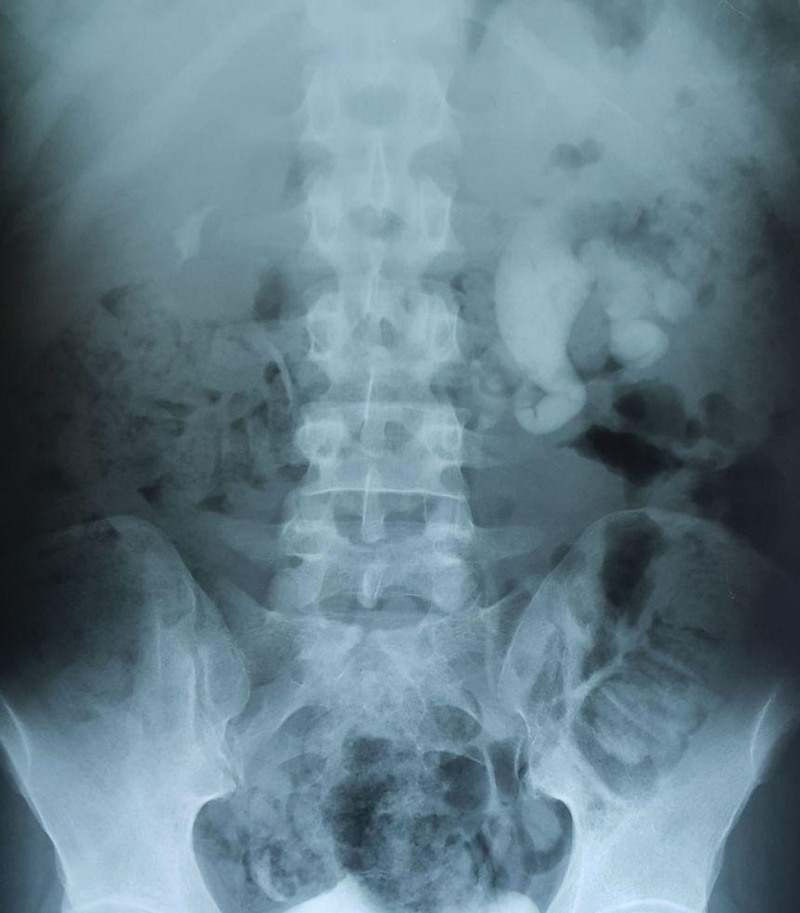
Intravenous pyelogram showed left-sided dilatation of the ureteropelvic junction and tortuosity of the proximal ureter indicative of obstruction at the L3-L4 disc space level.

Upon physical examination, no significant clinical findings were observed, such as tenderness at the costovertebral angle area, a palpable mass, or peripheral lymphadenopathy. Urinalysis and urine cytology showed no abnormal findings. Furthermore, the results of routine blood investigations were within the normal reference ranges, except for an elevated C-reactive protein level of 176 mg/L. The abdominal X-ray did not reveal any abnormal findings. Intravenous pyelogram performed with prone positioning demonstrated left-sided dilation of the pelvicalyceal system, along with dilation and tortuosity of the proximal ureter due to obstruction at the level of L3-L4 disc space (Fig. 1).

**Figure 2.**
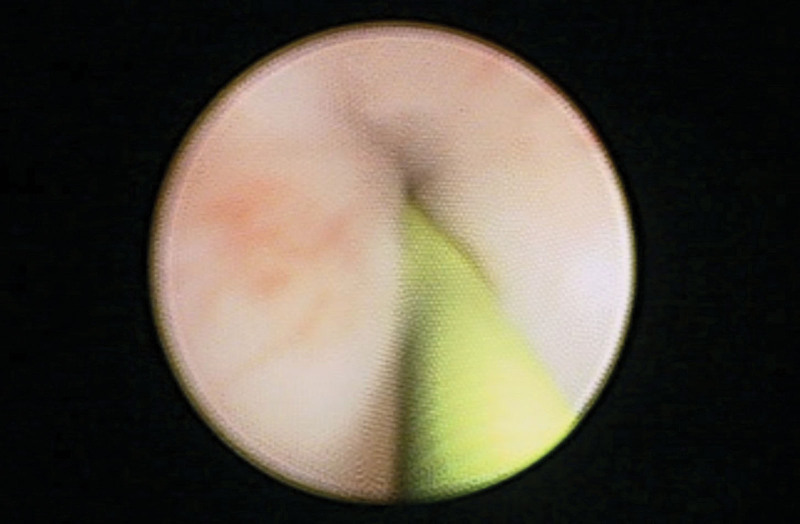
Ureteroscopy image while insertion of a double J stent showing luminal narrowing attributable to an extrinsic lesion.

**Figure 3.**
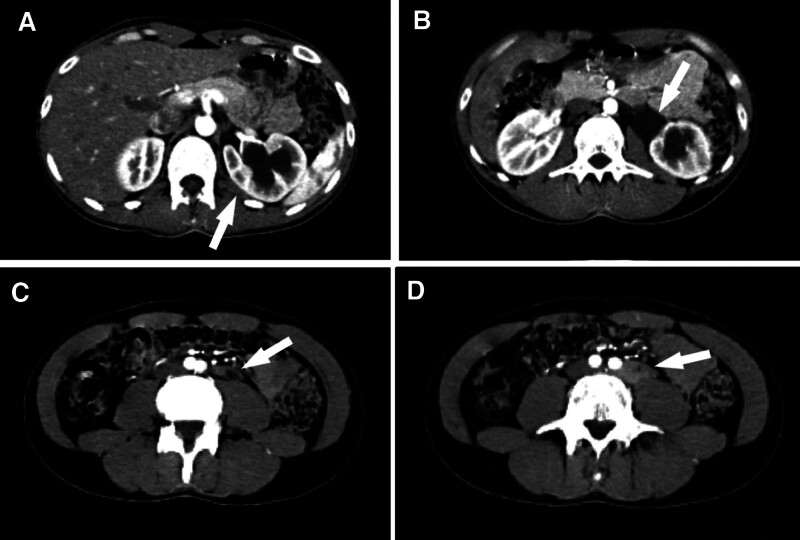
Axial contrast-enhanced computed tomography scans. (A) and (B) Hydroureteronephrosis of the left kidney with dilation of the pelvicalyceal system. (C) and (D) Homogenous lesion with uniform enhancement measuring 3.5 × 2 × 5.2 cm in the left retroperitoneum, adjacent to the left iliac artery, and encasing the ureter (indicated by arrows).

Following a contrast-enhanced computed tomography scan, grade 2 left hydroureteronephrosis was noticed, and a soft tissue mass measuring 3.5 × 2 × 5.2 cm in the greatest dimension was identified in the left retroperitoneum with a density of about 75 Hounsfield units. The lesion was found to be isodense with mild to moderate enhancement, adjacent to the common left iliac artery. It was encasing and compressing the ureter, leading to severe stenosis. Additionally, the mass was poorly separated from the left psoas major muscle (Figs. 2 and 3). However, no evidence of lymphadenopathy or metastatic lesion was observed.

**Figure 4.**
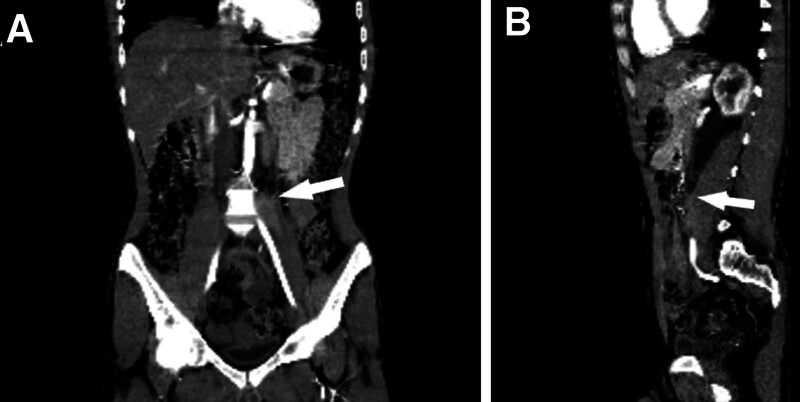
Contrast-enhanced computed tomography in the coronal section (A) and sagittal section (B) revealed a lesion obstructing the ureter at the level of the fourth vertebra, resulting in ureteral dilatation proximal to the obstruction (indicated by arrows).

Based on the previous findings, a ureteroscopy examination was subsequently indicated to further characterize the anatomical origin of the lesion. During the procedure, a severe narrowing of the ureter lumen was observed; however, no intrinsic obstructive lesion was noted, confirming the extrinsic origin of the lesion (Fig. 4). To maintain kidney functions, a double J catheter was successfully inserted to bypass the stenosis following several attempts.

As the imaging results were atypical, an extra-abdominal (retroperitoneal) tumor was suspected, and the patient was scheduled for exploratory surgery as it was challenging to make a definitive diagnosis and surgical intervention to treat the ureteral obstruction was necessary.

An exploratory laparotomy was performed with a paramedian incision. Upon gross examination of the surgical site, the tumor was found to have arisen from the connective tissue of the overlying fascia of the psoas major muscle. The lesion exhibited a firm texture, poor mobility, and a poorly circumscribed, grayish-white appearance.

The mass was abutting the common iliac artery and had encased and compressed the middle third of the ureter, leading to severe dilation of the proximal end. Using the double J catheter as a guide for ureteral identification, the left ureter was successfully isolated and preserved through careful dissection, starting from the lateral aspect. After complete isolation of the ureter, a meticulous, wide excision of the tumor was performed, resulting in the absence of macroscopic residual disease. No intraoperative complications were encountered, and the patient had an uneventful postoperative course before being discharged in good general condition on postoperative day 2.

**Figure 5.**
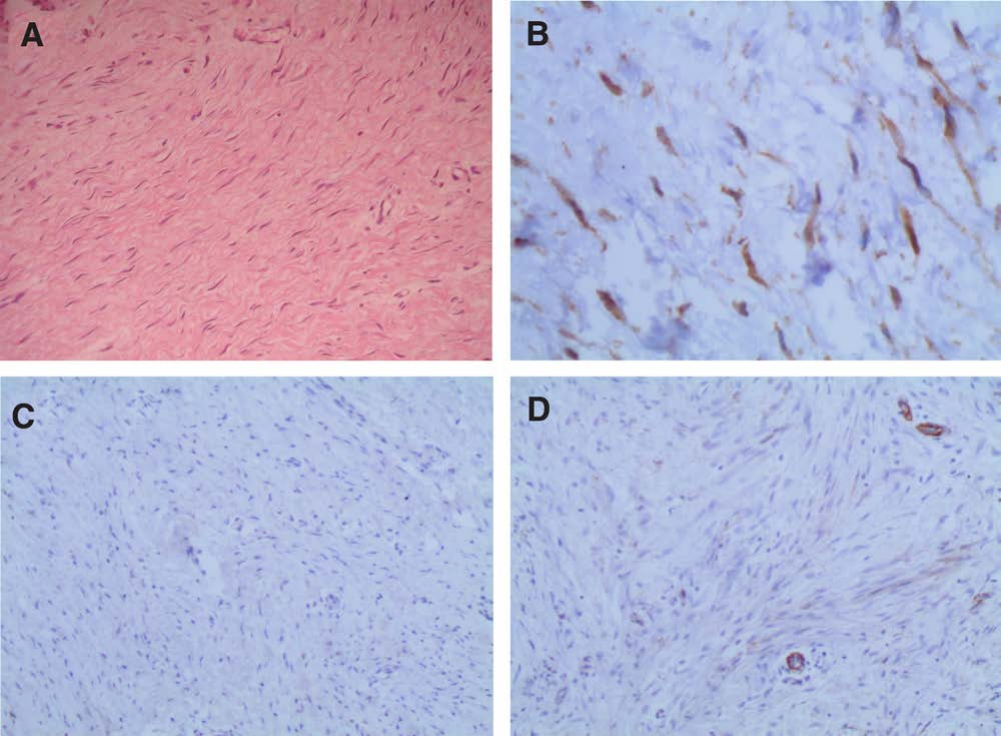
Histological findings of specimen. (A) Histopathological examination using hematoxylin and eosin stain showed the proliferation of spindle-shaped cells arranged within a collagenous background (×20 magnification). Immunohistochemical staining revealed: (B) positive staining for intranuclear β-catenin (×20 magnification), (C) negative staining for the S100 marker (×10 magnification), and (D) negative staining for the smooth muscle actin marker (×10 magnification).

On histopathological examination, the tumor comprised bland, uniform spindle-shaped fibroblasts arranged in broad, long, sweeping fascicles with a relatively collagenous background. There was no evidence of significant inflammatory cell infiltrates, nuclear atypia, or increased mitotic activity. An immunohistochemical study showed that the tumor cells were positive for B-catenin but negative for smooth muscle actin, S100, CD34, CD117, cytokeratin, and desmin, with Ki67 < 1% (Fig. 5).

Based on the combined pathology and immunohistochemistry results, the tumor was consistent with aggressive fibromatosis (AF) or DTF.

The double J stent was extracted in the third week after regression of the hydronephrosis, which was confirmed by US.

In the absence of established imaging guidelines for postoperative follow-up of DTF, the patient underwent periodic assessments after surgery. For the initial 12 months, abdominal US was performed every 3 months, along with magnetic resonance imagings every 4 months, revealing no evidence of recurrence. Subsequently, the patient had outpatient visits for the following 4 years, undergoing periodic abdominal US and comprehensive laboratory examinations. Throughout the 5-year follow-up period, he remained symptom-free with no evidence of local recurrence.

## 3. Discussion

DTF, also referred to as AF, is an extremely rare and benign neoplasm of the soft tissue that typically originates from fascial or musculoaponeurotic structures.^[3]^ It comprises only 0.03% of all newly diagnosed tumors and <3% of all soft tissue neoplasms.^[1]^ In 1982, Reitamo et al^[2]^ reported the incidence of AF in the Finnish population, estimating it to be 2.4 to 4.3 cases per million individuals per year. Despite the slow-growing and nonmetastatic nature of DTF, it exhibits a locally aggressive and unpredictable behavior with a high potential for recurrence even when wide surgical margins are performed.^[4,5]^ DTF can occur at any age but typically manifests in individuals aged 15 to 60 years, with a peak around 30 years.^[5]^ Moreover, there is a notable gender discrepancy, with the female-to-male ratio ranging from 2:1 to 5:1.^[2,6]^

Although the definitive etiology of DTF remains unclear, suggested risk factors include a history of trauma, family history of DTF, previous pregnancy, estrogen exposure, irradiation, genetic mutation (3’ adenomatous polyposis coli germline mutation), and FAP or Gardner syndrome.^[2,3,12,13]^ Our case is sporadic, with no previous personal or family history of FAP or abdominal trauma.

DTF can be clinically classified into 2 main types: The predominant type is sporadic in nature, and another type is familial and usually occurs in association with FAP, presenting as Gardner syndrome and accounting for approximately 3% to 16% of cases that tend to develop intra-abdominally.^[2,10,12,14,15]^

Compared to the general population, patients with FAP have an increased risk of developing DTF that is more than 800-fold higher, which makes the prevalence of DTFs in such patients range between 10% and 25%.^[9,12,16,17]^

This tumor demonstrates heterogeneous behavior, ranging from growth to stability or even regression without medical intervention, leading to varied clinical manifestations such as pain, swelling, and movement restrictions. These symptoms are primarily influenced by the tumor’s anatomical location, size, and relation to adjacent structures. For instance, such behavior may lead to abdominal compression of viscera, which can manifest as symptoms of intestinal, vascular, and ureteric obstruction, as well as neural involvement.^[3,4,7,9]^ However, in a considerable percentage of patients, DTFs remain asymptomatic and are discovered incidentally during routine medical examinations.^[3,7,8]^ In our case, despite the tumor causing unilateral extrinsic obstruction of the urinary tract and significant dilatation of the pelvicalyceal system, the patient remained asymptomatic. This suggests a gradual progression of the tumor over a long period of time. However, hydroureteronephrosis was discovered incidentally during a routine checkup following open-heart surgery for ASD.

DTF may manifest anywhere in the body,^[3]^ with their anatomical locations typically classified into extra-abdominal (extremities) abdominal cavity or wall and thorax comprising approximately 32%, 23%, 21%, and 15% of cases, respectively. Notably, extra-abdominal tumors carry a higher risk of recurrence.^[13]^ To the best of our knowledge, only 3 cases of DTF originating from the psoas major muscle have been documented,^[18–20]^ all presenting with symptoms. Among these cases, only 1 occurred in a male patient, and none exhibited hydronephrosis. Our patient represents the fourth documented case of DTF originating from the psoas major muscle in the English medical literature.

Histopathological examination, in addition to immunohistochemical staining, remains the gold standard for establishing a conclusive diagnosis of desmoid tumors. Microscopically, it consists of uniformly interspersed spindle-shaped cells surrounded by a dense collagenic matrix with positive staining for nuclear β-catenin, which, though significant for supporting the diagnosis, is not disease-specific.^[7,8]^

Optimal management of desmoid tumors requires collaborative efforts of a multidisciplinary team. There is a broad spectrum of treatment options available, such as radiation therapy, systemic therapy, and surgical excision. Systemic therapy includes hormone therapy (antiestrogen agents) nonsteroidal anti-inflammatory drugs, chemotherapy, and targeted therapies.^[5,9,21]^ In the past, surgical excision has been the conventional standard of treatment for desmoid tumors. However, its role as the go-to treatment is now controversial due to concerns about surgical tissue trauma, which contributes to tumor stimulation and increases morbidity, and the fact that some tumors exhibit spontaneous regression after careful monitoring. Consequently, the first-line approach is now a watchful waiting policy, especially in asymptomatic patients (watchful waiting policy). However, the treatment strategy should be personalized and determined by tumor growth behavior and associated complications.^[4,9–11]^ In our situation, a conservative approach was not feasible due to severe ureteral obstruction and significant pelvicalyceal system dilation. The case was managed by inserting a ureteric double J stent to alleviate the restriction of urinary flow. Subsequently, surgical intervention was carried out, and the tumor was resected after successful isolation of the ureter from the surrounding lesion.

Desmoid tumors demonstrate a propensity for local recurrence even following complete surgical excision, and within 5 years, up to 50% of patients may experience a relapse. Factors that are seemingly associated with recurrence include younger age, larger tumor size, extremity (extra-abdominal) tumor site, and margin status.^[13,21]^ Nonetheless, the significance of microscopic surgical margins has become a subject of considerable conflict within the medical literature. While achieving negative margins should be a surgical goal, recent studies have indicated that they do not consistently correlate with rates of recurrence.^[5,10,22,23]^

Regarding the optimal approach for postinterventional follow-up of patients with DTF, there is still no definitive consensus.^[24]^ However, after the excision of the tumor, the patient has been monitored for approximately 60 months and continues to be free of recurrence and in excellent health condition at the time of the preparation of this article.

## 4. Conclusion

The diagnosis and treatment of desmoid tumors remain challenging, particularly in cases where the tumor is incidentally discovered. In this report, we present a complex surgical case of a retroperitoneal desmoid tumor originating from the fascia of the psoas major muscle. Although the patient was asymptomatic, surgical intervention with exploratory laparotomy was a crucial step in function preservation of the left kidney. The encasement of the ureter by the tumor presented a potential risk of ureteral resection and subsequent ureteroureterostomy. However, the insertion of a double J stent facilitated the successful isolation of the ureter, allowing for safe tumor resection.

Fortunately, no intraoperative complications were encountered, and the patient remained recurrence-free and in good general condition during postoperative follow-up.

The patient was able to return to his daily activities without any difficulty following the treatment.

## Acknowledgments

The authors are grateful to Dr Hasan Haddad and Dr Rafiq Sarhel for their expert radiological evaluations. They also wish to express their sincere appreciation to Dr Mazen Haydar for his invaluable consultations in the patient management.

## Author contributions

**Conceptualization:** Abdulrahman Almjersah

**Data curation:** Abdulrahman Almjersah, Habib Olaisheh, Rabab Salloum

**Formal analysis:** Abdulrahman Almjersah, Habib Olaisheh

**Investigation:** Abdulrahman Almjersah, Habib Olaisheh

**Methodology:** Abdulrahman Almjersah, Habib Olaisheh, Emad Almjersah

**Project administration:** Abdulrahman Almjersah, Rabab Salloum, Emad Almjersah

**Software:** Abdulrahman Almjersah, Habib Olaisheh

**Validation:** Abdulrahman Almjersah, Rabab Salloum, Zuheir Alshehabi, Emad Almjersah

**Visualization:** Abdulrahman Almjersah, Habib Olaisheh

**Writing – original draft:** Abdulrahman Almjersah

**Writing – review & editing:** Abdulrahman Almjersah, Zuheir Alshehabi, Emad Almjersah

**Funding acquisition:** Habib Olaisheh, Emad Almjersah

**Resources:** Habib Olaisheh, Rabab Salloum

**Supervision:** Rabab Salloum, Zuheir Alshehabi, Emad Almjersah
